# The cone visual cycle and its disorders: insights from zebrafish

**DOI:** 10.3389/fnmol.2025.1730071

**Published:** 2025-11-21

**Authors:** Ioanna S. Koutroumpa, Stephan C. F. Neuhauss

**Affiliations:** Department of Molecular Life Sciences, University of Zurich, Zurich, Switzerland

**Keywords:** visual cycle, retina, retinoids (vitamin a derivatives), zebrafish, cone photoreceptor, vision

## Abstract

Continuous vision relies on the recycling of visual pigment chromophore, which is photoisomerized during the process of vision. In vertebrates, this recycling is mediated by a complex network of biochemical reactions distributed across different cell types referred to as the visual cycle. In this review, we outline both historical and recent findings on the visual cycle and its connection to outer retinal dystrophies. Particular emphasis is placed on the recycling of cone, rather than rod, visual pigments, and on the utility of the zebrafish (*Danio rerio*) as a model for such studies.

## The canonical visual pathway

**Figure 1:**
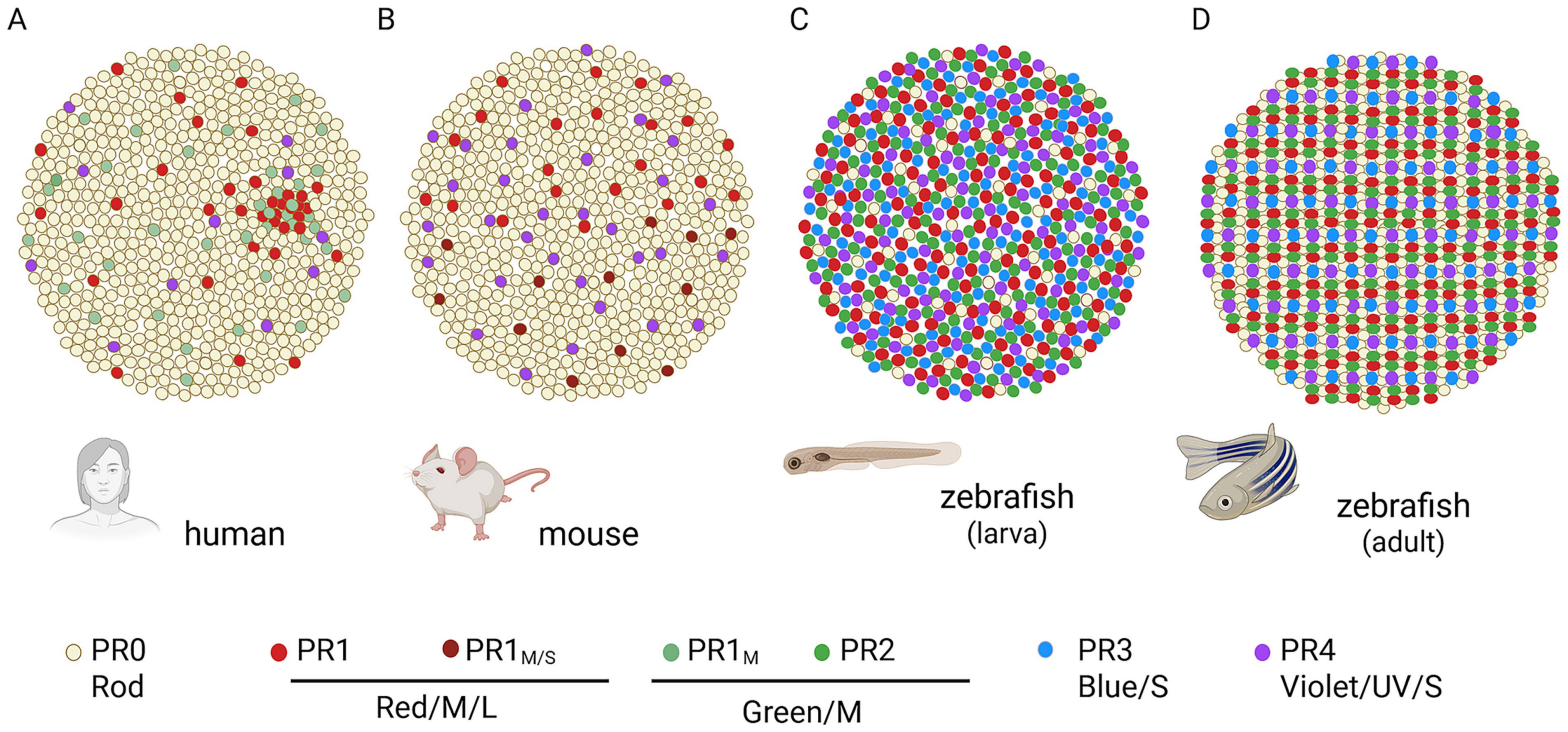
Photoreceptor mosaic organization in the human **(A)**, mouse **(B)**, and zebrafish **(C,D)** retina. The human retina is known to be trichomatic and rod-dominant with the macula and in the very center the fovea that has cone-only structure (Hofer et al., 2005). Mouse retina is dichromatic rod dominant that lacks macula but there is a central area that has a higher ratio of cones-to-rods (Applebury et al., 2000). Zebrafish has tetrachromatic vision and two distinct mosaic patterns: the larval one that has very few non-responsive rods and the recordings come from cones-only and the juvenile/adult mosaic that has physiologically responsive rods but is still cone dominant (Allison et al., 2010). Updated photoreceptor nomenclature of Baden et al. is used that is based on the physiological and molecular identity of the neurons and not the spectral properties of their opsins (Baden et al., 2025). Image *created in BioRender.*

The vertebrate retina consists of three neuronal layers, the Outer Nuclear Layer (ONL) consists of photoreceptor, the photosensitive neurons that convert the physical stimulus to a biological signal. Photoreceptors are divided into two groups; rods that contribute to dim-light or night vision, and cones that support bright-light or daylight vision. The classification of vertebrate rods and especially cones has long been debated due to species-specific differences in spectral sensitivity, absorption spectra, and opsin expression. Recently, Baden et al. proposed a revised photoreceptor nomenclature, as shown in Figure 1 (Baden et al., 2025).

The photoreceptor composition and topology in the retina are also species-specific, at least partially influenced by the visual ecology that the animal lives in. Photoreceptors contain the visual chromophore 11-cis retinal (11-cis-RAL), a derivative of vitamin A (retinal A1), that is covalently bound to opsins located in their outer segments (OS) of the photoreceptors. Upon illumination, 11-cis-RAL is converted to all-trans retinal (all-trans-RAL) that then detaches from its opsin (Wald, 1968). After reduction to all-trans retinol, this molecule is leaving the photoreceptor OS, and is recycled via a multistep process now known as the canonical visual cycle, before reentering the outer segments as 11-cis-RAL. Although 11-cis-RAL is the main prosthetic group of vertebrate opsins, some fish and amphibians also use 11-*cis* 3,4-didehydroretinal (A2 retinal) that has been associated to turbid or red light dominated environments (Corbo, 2021; Hagen et al., 2023). A2 can be taken up by both photoreceptor types and is most commonly found in amphibians and freshwater fish (Allison et al., 2004; Enright et al., 2015). Interestingly, zebrafish can shift opsin binding from A1 to A2 by thyroid hormone treatment (Allison et al., 2004).

In 1876 Friedrich Boll was the first to observe light-induced bleaching and regeneration of the “red” pigment (now known as rhodopsin) in the frog retina, noting that the process can be reversed when the tissue was kept in the dark (Boll, 1877). Subsequently, Franz Kühne demonstrated that this regeneration required interaction between the retina and another tissue, the retinal pigment epithelium (RPE) (Kühne, 1878). Over the span of two decades, George Wald and his colleagues elucidated what is now known as the retinoid recycling pathway or visual cycle (Dowling, 1960; Wald and Brown, 1956), a discovery that earned Wald the Nobel Prize in Physiology or Medicine in 1967.

**Figure 2:**
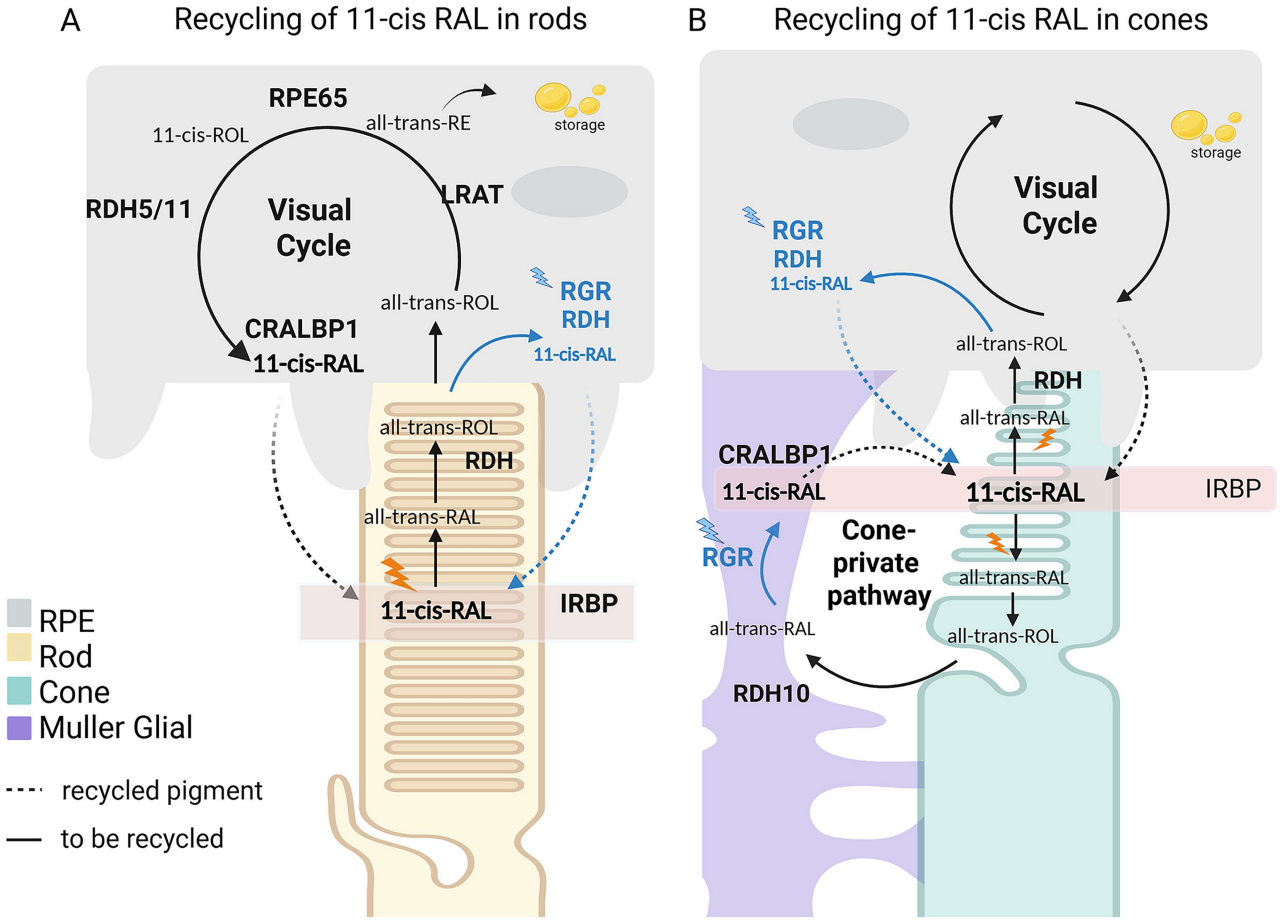
Model for recycling of 11-cis retinal (RAL) for rods **(A)** and cones **(B)** in vertebrates. **(A)** The RPE-mediated visual cycle provides both rods (yellow) and cones (green) with recycled 11-cis-RAL (in dashed arrows). Upon excitation 11-cis-RAL is photoconverted to all-trans RAL that is converted by an all-trans retinol dehydrogenase 8 or 12 (RDH8/12)to all-trans ROL that is then shuttled to the RPE (in grey) and is subsequently converted to all-trans retinylesters (REs) by lecithin retinol acyltransferase LRAT. These all-trans REs are either stored in lipid droplets or isomerized by RPE65 to 11-cis-ROL and finally converted to 11-cis-RAL by another cis retinol dehydrogenase 5 or 11 (RDH5/11). 11-cis-RAL binds to CRALBP1 to prevent photodegradation and is supplemented at any time to the photoreceptor outer segments. Assisted transportation retinoids between the RPE and the photoreceptors is mediated by the IRBP. Additionally, RGR re-isomerizes all-trans-ROL in the RPE by absorbing blue light, independent of the visual cycle. **(B)** Cone photoreceptors have an additional pathway mediated by Müller Glia cells (in purple) with some still unidentified components. Among the identified proteins are RGR photoisomerase, CRALBP1 retinoid binding protein, RDH10 dehydrogenase, nonetheless the precise steps are not defined. Image *created in BioRender.*

The RPE-mediated visual cycle regenerates 11-cis-RAL for both rod and cone photoreceptors (Figure 2). Upon light absorption, 11-cis-RAL is photoisomerized to all-trans-RAL, which is reduced by all-trans retinol reductase 8 (RDH8) to all-trans-retinol (all-trans ROL) in the photoreceptor outer segments or by all-trans retinol reductase 12 (RDH12) in the inner segment (Parker and Crouch, 2010). This retinoid is transported to the RPE, where lecithin retinol acyltransferase (LRAT) esterifies it to all-trans-retinyl esters (REs) (Sears and Palczewski, 2016). These REsare either stored in lipid droplets or directly isomerized by the bona fide isomerase RPE65 (retinal pigment protein of 65kD) into 11-cis-retinol (11-cis-ROL) (Kiser, 2022), which is subsequently oxidized by either cis-retinol dehydrogenase 5 or 11 (RDH5/11) to regenerate 11-cis-RAL. To prevent photodegradation and enable solubilization in an aqueous environment, 11-cis-RAL binds to cellular retinaldehyde-binding protein 1 (CRALBP1), which stabilizes 11-cis-RAL until it is shuttled to the photoreceptor outer segments (Morimura et al., 1999a; Saari and Crabb, 2005). The assisted retinoid transportation is mediated by Interphotoreceptor retinoid-binding protein (IRBP). In parallel to the canonical visual cycle, retinal G protein–coupled receptor (RGR) functions as a light-driven photoisomerase in the RPE, converting all-trans-ROL to 11-cis-ROL upon blue-light absorption (Figure 2, steps in blue) (Ruddin et al., 2025). The discovery of a second RPE-independent pathway and the cone specific recycling of 11-cis-RAL was widely accepted only in the early 2000s (Fleisch and Neuhauss, 2010; Mata et al., 2002b; Muniz et al., 2007; Wang and Kefalov, 2009, 2011).

## A cone specific inner-retinal recycling pathway

The existence of this second intra-retinal recycling pathway was already suggested by electrophysiological recordings in frogs in the 1970s by Goldstein and Wolf (Goldstein, 1968, 1967; Goldstein and Wolf, 1973; Hood and Hock, 1973). Additional evidence was provided by Jones and colleagues in 1989 when they showed that rod and cone sensitivity was restored by supplementing 11-cis-RAL, but only cones recover by supplying 11-cis-ROL, implying different recycling pathways (Jones et al., 1989). A decade later *in vitro* chicken Muller glia (MG) cell culture experiments demonstrated that these cells are capable of synthesizing 11-cis-ROL (Das et al., 1992). Subsequently, Mata, Muniz and their colleagues, confirmed in *in vivo* experiments the existence of an additional cone-specific, RPE-independent pathway, using cone-dominant chicken (Mata et al., 2005, 2002a; Muniz et al., 2007) and ground squirrel (Mata et al., 2002b) retinas. Moreover, the chemical ablation of Muller glia cells in mouse, salamander, and primate retinas by L-*α*-aminoapidic acid (L-α-aa) initially affected cone recovery and the supplementation with 11-cis-ROL rescues cone responses (Wang and Kefalov, 2009), providing additional evidence for an RPE independent recycling pathway. Zebrafish has also provided valuable information to the field with their cone dominant retina (Collery et al., 2008; Fleisch et al., 2008; Schonthaler et al., 2007). It is now well established that cones also rely on a second recycling pathway involving MG cells, where RGR is considered the main isomerase, while the once proposed candidate dihydroceramide desaturase-1 (DES1) was later ruled out (Mata et al., 2002b; Kaylor et al., 2013; Kiser et al., 2019; Wang and Kefalov, 2011; Tworak et al., 2023). Similar to the canonical visual cycle, CRALBP1 binds to 11-cis-RAL, 11-cis retinol oxidase 10 (RDH10) is the current candidate retinol dehydrogenase, even though the knockout mouse retina had no severe defects(Xue et al., 2017), and a retinyl ester hydrolase, presumably multifunctional-O-acyltransferase (MFAT) have been identified as components of the cone private path. (Kaylor et al., 2015; Widjaja-Adhi et al., 2022) (see Figure 2B) However, the exact biochemical steps of this cone-specific pathway have yet to be fully delineated.

Even though the canonical visual cycle pathway is the shared pathway for cones and rods, the cone private pathway is believed to be ancestral. Cones are the ancestral vertebrate photoreceptors types with rods appearing at the base of the vertebrate radiation [reviewed by Hunt and Collin (Hunt and Collin, 2014)]. Phylogenetic studies between vertebrate and invertebrate outgroups showed that the ancestral rod opsin RH duplicate evolved into the jawed vertebrate rhodopsin Rh1 (Collin et al., 2003). In the basal vertebrate lamprey, orthologs of RPE65 and LRAT are present and exhibit enzymatic activities consistent with retinoid isomerization and esterification, respectively, similar to those observed in other vertebrate visual system cycles (Poliakov et al., 2012; Vopalensky et al., 2012). In contrast, members of the cephalochordate lineage, the sister group to vertebrates, possess a central eye composed of a mosaic of rod-like and cone-like photoreceptors associated with pigmented epithelial cells, yet lacking functional orthologs of RPE65 and LRAT (Poliakov et al., 2012). This suggests that 11-cis-RAL was initially recycled via the cone-private pathway.

The significance of the dynamics between the canonical visual cycle and the cone private pathway becomes apparent when light is taken into account. Rods are saturated under bright light conditions and therefore require maximum supply of retinoids. Cones have concomitantly a high demand for retinoids that may exceed recycling resources due to direct competition with rods, necessitating a cone private pathway (see Figure 2B). Thus, the species’ habitat and their retina composition (ratio rod-cone photoreceptors) is expected to affect the interplay between these two recycling pathways in a light dependent manner.

Defects in the recycling of 11-cis-RAL results in accumulation of all-trans RAL, one of the initial steps leading to photodamage (Maeda et al., 2012). Non-recycled all-trans-RAL is photoconverted to toxic retinoid by-products, with A2E being the most prominent one (Kim and Sparrow, 2021; Parish et al., 1998; Sparrow et al., 2010, 2003). Such toxic derivatives are enclosed in lipid droplets in the RPE called lipofuscin. These cause chronic oxidative stress and inflammation that result in photoreceptor degeneration and subsequent retinal detachment (Boyer et al., 2012; Feeney, 1978; Feldman et al., 2022; Kennedy et al., 1995; Wing et al., 1978). Depending on the underlying mutation, the disease may initially affect rods and subsequently cones, leading to rod–cone dystrophies such as autosomal or recessive Retinitis Pigmentosa (RP) and early-onset Leber congenital amaurosis (LCA) (Bernal et al., 2003; Fahim et al., 1993; Kumaran et al., 2017, 1993; Lima de Carvalho et al., 2020; Maeda et al., 2009; Morimura et al., 1999b; Sarkar et al., 2021). Defects in visual pigment recycling can secondarily affect cone photoreceptors in the human macula, leading to their degeneration and resulting in age-related macular degeneration (AMD), which typically manifests after the fifth decade of life. (Chen et al., 2012; Deng et al., 2021; Maeda et al., 2012; Somasundaran et al., 2020).

Despite more than a century of research on 11-cis-RAL recycling, and numerous comprehensive reviews on the canonical visual cycle, the cone-specific pathway and retinoid biochemistry, important gaps in our knowledge remain. The interaction between the canonical RPE-dependent visual cycle and the cone-specific MG mediated pathway remains incompletely defined, particularly regarding the contribution of the latter to cone physiology.

## How do studies in a cone-dominant retina contribute to our knowledge in rod-dominant retina species?

Most studies of visual pigment recycling were conducted using the retinas of rod-dominant mammalian and amphibian species. The salamander retina, with its large photoreceptors, was the first preparation to enable long-lasting electrophysiological recordings at single-cell resolution during light and dark adaptation (Wang et al., 2009). The bovine retina is large and readily accessible post mortem, making it well suited for biochemical studies. It has been widely used for retinoid analyses and as an *in vitro* system to study the RPE retinoid pool through microsome formation (Mata et al., 2002b; Zhang et al., 2019). These investigations were subsequently extended to the mouse retina, an established mammalian model amenable to genetic manipulation, leading to the generation of numerous visual cycle disease models used to study these pathways (Batten et al., 2004; Driessen et al., 2000; Liou et al., 1991; Redmond et al., 1998; Saari et al., 2001).

Light conditions, visual ecology and species-specific behavior influence the way that rods and cones recycle their visual pigments (Albalat, 2012; Guido et al., 2022; Hagen et al., 2023; Hankins et al., 2014; Musilova et al., 2021). Given the differences in retinal mosaics among zebrafish, mice, and humans (Figure 1), as well as variations in their rod-to-cone ratios (Table 1), the interplay between the canonical visual cycle and the cone-specific retinoid pathway is likely to differ substantially across species. Consequently, several models describing these mechanisms have been revised over the past decade. A prominent example illustrating the importance of species-specific context is the debated role of RGR (Morshedian et al., 2019; Radu et al., 2008; Tworak et al., 2023; Wenzel et al., 2005; Zhang et al., 2019).

**Table 1:** Cone: rod ratio for model organisms in vertebrate ophthalmology.

**Species**	**Fovea?**	**Cone: rod ratio**	**% Cones**
Human	Yes	~1:20	~5%
Macaca monkey (diurnal primate)	Yes	~1:20	~5%
Mouse	No	~1:30	~3%
Namaqua rock mouse (*Micaelamys namaquensis* – nocturnal rodent)	No	~1:12.4	~7%
Four-striped field mouse (*Rhabdomys pumilio* – diurnal/crepuscular rodent)	No	~1:1.23	~45%
Hamster	No	~1:32	~3%
Ground squirrel	No	~24:1	~96%
Cow (Bovine)	No	~1:12	~8%
Chicken	No	~3:2	~60%
Salamander/Frog	No	~2:1	~35%
Zebrafish larva	No	~23:2	~92%
Zebrafish juvenile/adult	No	~3:2	~60%

List of the most commonly used species in vision biology and their respective cone-to-rod ratio and the cone percentage in their retina mosaic (Allison et al., 2010; Wang and Kefalov, 2011). Primate retinas with a fovea consist of 100% cones.

Although the human retina is rod dominant, high visual acuity and color vision depend on the cones in the densely packed central macula (fovea), which accounts for only about 0.02% of the total retinal area (Kolb et al., 1995) (Figure 1A). Since the human retina is largely only accessible to non-invasive experiments, such as optical coherence tomography (OCT) or electroretinography (ERG), researchers have to rely on suitable model organism to study retinal metabolism. Because most models for studying pigment recycling are rod dominant, the scarcity of cones in these systems may have hindered accurate interpretation of cone-specific phenotypes caused by impaired pigment regeneration (Figure 1B). The cone dominant models that have been used to approach cone physiology such as the chicken and ground squirrel retina contributed immensely to record cone responses and assess the visual pigment recycling as mentioned above. However, these systems are not suitable for genetic approaches.

This is precisely why zebrafish serve as an excellent model for studying cone visual pigment recycling. During larval stages, the zebrafish retina is almost exclusively cone-based, with measurable ERG responses detectable from 4 days post-fertilization onwards (Bilotta et al., 2001). Allison et al. demonstrated that the larval retina contains ~92% cones, with only a few rods that do not contribute to vision (Figure 1C). At 15 days post-fertilization the retina begins to transition into a mixed mosaic (Figure 1D) that remains cone dominant (~60%) throughout life (Allison et al., 2010; Saszik et al., 1999). Ecologically, zebrafish are adapted to diurnal vision and there are many well-established genetic models for numerous human ocular diseases (Gestri et al., 2012; Rosa et al., 2023). Their powerful genetic toolbox enables targeted knockouts of genes of interest, with phenotypes assessable by histology and optical non-invasive OCT (Bailey et al., 2012; Bell et al., 2016). Visual function can be quantified both electrophysiologically via whole-field ERGs and single-photoreceptor recordings (Bilotta et al., 2001; Makhankov et al., 2004; Nelson and Connaughton, 1995; Saszik et al., 1999; Sato and Kefalov, 2025) and behaviorally by using assays such as the optokinetic response (OKR), visuomotor response (VMR), and optomotor response (OMR) (Baier, 2000; Brockerhoff et al., 1995; Neuhauss et al., 1999; Neuhauss, 2003). For retinoid analysis, ocular extracts can be assessed by HPLC, and modern techniques even allow *in situ* imaging of retinoid fractions within the RPE, a method not feasible in humans (Babino et al., 2015). Collectively, these tools enable a comprehensive and detailed analysis of the cone-dominant zebrafish retina, providing valuable insights for both comparative and translational vision research.

## Zebrafish: illuminating the cone visual cycle

Studies in the zebrafish have expanded our understanding of visual pigment recycling. The first findings focused on the visual cycle isomerase RPE65 with the knockdown of RPE65a and pharmacological inhibition of RPE65. Both approached led to the conclusion that there was a reduction in 11-cis-RAL leading to impaired rod function while cone-driven visual behavior remains largely intact. These results support the notion of an RPE65-independent pathway for cone chromophore regeneration in zebrafish (Schonthaler et al., 2007). Two additional paralogs of RPE65 have been described in zebrafish. While *rpe65b* is not expressed in the retina (Schonthaler et al., 2007), *rpe65c* (really a tandem duplicate of *rpe65b*, more aptly named *rpe65ba* and *rpe65bb*) was reported to be expressed in MG cells (Takahashi et al., 2012, 2011). The authors demonstrated isomerohydrolase activity in cell lysates. These results are still awaiting confirmation by other researchers. Recently an inconclusive report on *rpe65a* knockdown larvae was published reporting a surprisingly high lethality (Mirzaei et al., 2024).

The two paralogs of *cralbp1*, also referred to retinaldehyde-binding protein1 (*rlbp1*) were identified in zebrafish, leading to an interesting scenario of subfunctionalization (Fleisch et al., 2008). *rlbp1a* is expressed in the RPE, while *rlbp1b* is localized to MG cells. The mouse ortholog is expressed in both cell types. Both single knockdown animals displayed reduced saccades frequency in the OKR assay (Collery et al., 2008). Moreover, the knockdown of either paralogue reduces 11-cis-RAL and combined knockdown effect is additive, along with the reduction of their b-wave amplitude in response to light stimulation (Fleisch et al., 2008). Lastly, *rlbp1a* (RPE-CRALBP1a) knockout had reduced 11-cis-RAL, impaired scotopic and photopic ERG responses and morphological analysis revealed REs accumulation as enlarged retinosomes in the RPE of these mutants closely modeling human RLBP1 disease (Schlegel et al., 2022). The lack of an apparent phenotype for the MG-expressed *rlbp1b* KO line has been attributed to functional compensation by the 11-cis-RAL recycling machinery. These results collectively proved that both RPE and MG cell pathways contribute to cone pigment regeneration. In the RPE-deficient *cralbp1* knockout zebrafish, human RLBP1 variants are screened via CRISPR/Cas9 knock-in. However, expression of wild-type human CRALBP failed to rescue the phenotype, as determined by the dim-light VMR assay. Notably, key visual-cycle and vitamin A handling proteins (e.g., Lrat, Rdh5, Stra6, Rpe65a, Rgr) were upregulated in knock-out eyes, consistent with compensatory responses to reduced 11-cis retinoids (Fehilly et al., 2025).

RDH12, a retinol dehydrogenase expressed in the photoreceptor inner segment, is another visual cycle component that was also studied in zebrafish. In the *rdh12* mutants mislocalized rhodopsin was detected in inner segments, which implies impaired opsin trafficking and is an early sign of photoreceptor degeneration. Moreover, the mutant RPE had enlarged phagosomes, indicative of defects in outer segment phagocytosis and disrupted retinoid recycling. Interestingly, the oxidative-stress related superoxide dismutase *sod2* gene expression was significantly reduced in mutant retinas, indicative of beginning neurodegeneration (Sarkar et al., 2021).

Babino and colleagues reported that the 11-cis-RE pools in the RPE provide a reserve for visual pigment storage in cone-dominant retinas that they innovatively visualized with 2-photon microscopy. Pharmacological blockage of the canonical visual cycle or by eliminating rods in the zebrafish larva proved that these 11-RE pools sustain cone vision in varying light conditions (Babino et al., 2015). Pharmacological and genetic perturbations were also used in the context of visual pigment recycling to investigate photopic vision. They recorded OKR of zebrafish larvae that had been treated with Emixustat, an RPE65 inhibitor, or fenretinide, which inhibits the retinol binding protein (RBP4) and DES1, and subsequently supplied with 9-cis-retinal, which was able to rescue the inhibitor-induced defects (Ward et al., 2020). Their assay, performed in a cone-dominant retina, revealed potential side effects on photopic vision. They further demonstrated that RPE65-mediated isomerization is essential for the immediate response to light, whereas sustained vision under bright light can be supported by photoisomerization, now known to be mediated by RGR.

## Conclusion

Within just a few decades of extensive research, significant progress has been made in understanding pigment recycling in cone photoreceptors. However, important gaps in knowledge remain. Key questions still to be addressed include (a) the identification of all components of the cone-specific pathway and its intermediates (b) the dynamics between the two pathways in varying light conditions and (c) the impact of defects in the cone-private pathways in the physiology of cones and rods.

The zebrafish offers unique advantages to bridge this gap of knowledge in cone pigment recycling. As a well-established model organism, it provides a state-of-the-art genetic toolkit and two cone-dominant retinal mosaics. The larval mosaic (Figure 1C) consists exclusively of physiologically responsive cones, whereas in the juvenile and adult mosaic (Figure 1D), rods are present but cones remain predominant. These features, together with the potential for high-throughput drug screening, make zebrafish as a powerful system to resolve current discrepancies in the cone physiology. There are plenty of visual pigment recycling drugs in clinical trials tested solely in rod-dominant species for RP. These experiments should also be conducted in cone-dominant retinas to increase their translational relevance to the human retina. We suggest additional screening using zebrafish RP models for assessment of these visual pigment recycling inhibitors/modulators (Swigris et al., 2025; Zaluski et al., 2025).

Ultimately, findings from zebrafish cone-dominant retina along with other models from different species will collectively help us dissect the dynamics of the visual pigment recycling and get a better understanding of cone physiology.
